# Integrated analysis of microRNA and mRNA expression and association with HIF binding reveals the complexity of microRNA expression regulation under hypoxia

**DOI:** 10.1186/1476-4598-13-28

**Published:** 2014-02-11

**Authors:** Carme Camps, Harpreet K Saini, David R Mole, Hani Choudhry, Martin Reczko, José Afonso Guerra-Assunção, Ya-Min Tian, Francesca M Buffa, Adrian L Harris, Artemis G Hatzigeorgiou, Anton J Enright, Jiannis Ragoussis

**Affiliations:** 1 The Wellcome Trust Centre for Human Genetics, University of Oxford, Roosevelt Drive, Oxford, United Kingdom; 2 EMBL—European Bioinformatics Institute, Wellcome Trust Genome Campus, Hinxton, United Kingdom; 3 Henry Wellcome Building for Molecular Physiology, University of Oxford, Roosevelt Drive, Oxford, United Kingdom; 4 Institute of Molecular Oncology, Biomedical Sciences Research Center “Alexander Fleming”, 34 Fleming Street, Vari 16672, Greece; 5 Cancer Research UK Molecular Oncology Laboratories, Weatherall Institute of Molecular Medicine, University of Oxford, John Radcliffe Hospital, Oxford, United Kingdom; 6 Department of Biochemistry, Faculty of Science, King Abdulaziz University, Jeddah, Saudi Arabia; 7 Present Address: McGill University and Genome Quebec Innovation Centre, 740 DR Penfield Ave, Montreal H3A 0G1, Canada

**Keywords:** MicroRNA, Hypoxia, HIF, Transcription factor, Gene regulation

## Abstract

**Background:**

In mammalians, HIF is a master regulator of hypoxia gene expression through direct binding to DNA, while its role in microRNA expression regulation, critical in the hypoxia response, is not elucidated genome wide. Our aim is to investigate in depth the regulation of microRNA expression by hypoxia in the breast cancer cell line MCF-7, establish the relationship between microRNA expression and HIF binding sites, pri-miRNA transcription and microRNA processing gene expression.

**Methods:**

MCF-7 cells were incubated at 1% Oxygen for 16, 32 and 48 h. SiRNA against HIF-1α and HIF-2α were performed as previously published. MicroRNA and mRNA expression were assessed using microRNA microarrays, small RNA sequencing, gene expression microarrays and Real time PCR. The Kraken pipeline was applied for microRNA-seq analysis along with Bioconductor packages. Microarray data was analysed using Limma (Bioconductor), ChIP-seq data were analysed using Gene Set Enrichment Analysis and multiple testing correction applied in all analyses.

**Results:**

Hypoxia time course microRNA sequencing data analysis identified 41 microRNAs significantly up- and 28 down-regulated, including hsa-miR-4521, hsa-miR-145-3p and hsa-miR-222-5p reported in conjunction with hypoxia for the first time. Integration of HIF-1α and HIF-2α ChIP-seq data with expression data showed overall association between binding sites and microRNA up-regulation, with hsa-miR-210-3p and microRNAs of miR-27a/23a/24-2 and miR-30b/30d clusters as predominant examples. Moreover the expression of hsa-miR-27a-3p and hsa-miR-24-3p was found positively associated to a hypoxia gene signature in breast cancer. Gene expression analysis showed no full coordination between pri-miRNA and microRNA expression, pointing towards additional levels of regulation. Several transcripts involved in microRNA processing were found regulated by hypoxia, of which *DICER* (down-regulated) and *AGO4* (up-regulated) were HIF dependent. DICER expression was found inversely correlated to hypoxia in breast cancer.

**Conclusions:**

Integrated analysis of microRNA, mRNA and ChIP-seq data in a model cell line supports the hypothesis that microRNA expression under hypoxia is regulated at transcriptional and post-transcriptional level, with the presence of HIF binding sites at microRNA genomic loci associated with up-regulation. The identification of hypoxia and HIF regulated microRNAs relevant for breast cancer is important for our understanding of disease development and design of therapeutic interventions.

## Background

Hypoxia plays an important role in cancer, where the oxygen supply can be affected by tumour growth and subsequent alteration of the blood vessel network, leading to fatal consequences such as cell resistance to certain drugs and metastasis (reviewed in [1]). Under low cellular oxygen levels, the hypoxic inducible family of transcription factors (HIF) drive a complex transcriptional response that affects several biological processes relevant for the fate of the cells under these conditions, including glycolysis, angiogenesis and apoptosis [2]. HIF transcription factors are heterodimers composed of an alpha and a beta subunit. There are three HIF-alpha subunits, of which HIF-1α and HIF-2α have been shown to have important regulatory roles in the hypoxia response. HIF-alpha protein levels are regulated by the Von Hippel Lindau (VHL) protein which mediates the ubiquitination of HIF-alpha by specifically recognising and binding to two prolyl-hydroxylated residues, resulting on a rapid elimination of HIF-alpha. HIF-alpha prolyl hydroxylation is catalysed by three homologous 2-oxoglutarate dependent dioxygenases, PHD1, PHD2 and PHD3. Further control of HIF-alpha expression is performed by another dioxygenase: Factor Inhibiting HIF 1 (FIH-1). FIH-1 catalyses the formation of a specific hydroxyasparaginyl residue in HIF-alpha that reduces its binding to the transcriptional coactivator p300 (for a review see [3,4]). Since the hydroxylation of HIF-alpha residues by PHDs and FIH is oxygen dependent, HIF-alpha is able to escape VHL recognition and further proteasomal degradation under hypoxic conditions, bind to p300 and HIF-beta and induce transcriptional changes that also affect microRNAs.

MicroRNAs are small noncoding RNA oligonucleotides (~22 nucleotides) that regulate gene expression at post-transcriptional level. Their genomic organisation is heterogeneous. For instance approximately half of the microRNAs identified in mammals are found in intergenic regions whereas the other half are encoded within the open reading frame of other genes [5,6]. Nevertheless some microRNAs belonging to this last group have their own promoter. In addition microRNAs can be transcribed alone or in polycistronic units [7]. Independent of their origin, all microRNA transcripts undergo a complex process of biogenesis which takes part in different cellular compartments and involves many proteins. Briefly, first a microRNA containing transcript (pri-miRNA) is cleaved in the nucleus by a complex lead by DROSHA and DGCR8, generating 70-nucleotide long RNA molecules known as precursor microRNAs (pre-miRNAs). Pre-miRNAs are transported by EXPORTIN 5 from the nucleus to the cytoplasm where they are cleaved by DICER/TRBP complex, producing a 22 nucleotide microRNA duplex. This microRNA duplex is then loaded into the RNA-induced silencing complex (RISC) where ARGONAUTE (AGO) proteins have a key role on the recruitment of microRNAs and their mRNA targets. Finally one strand from the duplex mediates the regulation of target mRNAs by impairing their translation and/or by promoting their degradation. In most cases, there is a preference for the selection of one of the strands of the microRNA duplex in the RISC complex since both strands are detected at very assymetric levels, (for a review see [8]). Most of the earlier studies have focused on the most abundant strand (usually 5p), whereas the less abundant strand (also known as miRNA*) is considered to be degraded. However evidence is growing that miRNA* can actually be functional by regulating specific target mRNA [9,10]. Indeed, a mature miRNA is now designated with the suffix “-3p” or “-5p” depending on the originating strand and we have used the same nomenclature in this work.

Several studies have been performed in cancer cell lines, endothelial cell lines and primary human cytotrophoblasts that have identified microRNAs regulated by hypoxia [11-20]. Most of these studies were done using microarray or Real time PCR (qPCR) based platforms, thus limiting the findings to a set of microRNAs. The overlap between the microRNAs found significantly regulated in these studies is low, probably due to the high variability generated by the fact of using different cell types, different experimental conditions and different technologies for measuring microRNA expression. The role of HIF in the regulation of microRNA expression has not been deeply investigated and only for a few microRNAs there is some experimental evidence that HIF is directly involved in the regulation of their expression under hypoxia [16,21-23]. Also some elements of the microRNA processing pathway have been recently reported to be regulated under hypoxia [14,18,24], but the direct involvement of HIF through DNA binding remains mostly unknown. The first screenings for hypoxia regulated microRNAs in breast cancer and other solid tumours, suggested that hypoxia could be a key factor in microRNA modulation in cancer [16]. Moreover, some hypoxia regulated microRNAs have been shown to have functional roles that are of great relevance in cancer. Therefore, it is important to shed more light in this area.

Our aim is to investigate the regulation of microRNA expression by hypoxia, with a special focus in the role of HIF in this process. We have chosen the breast cancer cell line MCF-7 as a system because we have already shown that the HIF-1α and HIF-2α proteins are present and stabilized across different hypoxia time points, starting at 16 h, and we have characterised the role of both HIF-1α and HIF-2α in the regulation of gene expression in this system [25,26]. We have performed a comprehensive study, by integrating microRNA expression, gene expression and HIF-1α and HIF-2α chromatin immunoprecipitation (ChIP) data from the same breast cancer cell line MCF-7. We have used the latest next generation sequencing technology as well as established microarrays to generate microRNA expression profiles from a three point time course in hypoxia (16 h, 32 h and 48 h). We present here microRNAs that are found consistently regulated at different hypoxia time points, including novel hypoxia related microRNAs. Moreover, we have used gene expression profiles to assess the relationship between microRNA and host gene for the microRNAs located in intragenic regions and HIF-1α and HIF-2α ChIP-seq data to identify binding sites close to microRNAs. Finally we also used these data sets to investigate the expression of the genes involved in the microRNA processing pathway and the involvement of HIF in their regulation.

## Results

### Profiling the population of microRNAs in hypoxic MCF-7 cells by next-generation sequencing

The total number of sequencing reads obtained was in the range between 32 and 35 million reads per sample. After filtering, sequencing reads with a length between 18 and 26 nucleotides represented 75.2-87.5% of total number of reads obtained initially (Additional file 1: Table S1). On average, 99% of these reads (98.9-99.5%) were mapped to the human genome using Bowtie, from which 75-85.9% had a unique mapping position (Additional file 1: Table S1). Most of the reads mapped to annotated regions (98-98.9%), and as expected the vast majority of them matched genomic regions allocating microRNAs (94.5-96.1% of reads mapped to annotated regions). The residual fraction of mapped reads not corresponding to microRNAs was distributed among a miscellanea of annotated regions including other RNA and small RNAs species, protein coding exons, pseudogenes and repeat molecules (Additional file 1: Table S1).

We were able to identify 502 microRNAs consistently across all conditions in MCF-7 cells (Additional file 1: Table S2). Among them, 412 microRNAs had strand (-5p/-3p) annotations with 53.2% of 5p and 46.8% of 3p-microRNAs. We found that 184 microRNAs were from only one strand (either-5p or-3p), so there were no detectable traces of the other hairpin arm and 114 microRNAs have both the 5p and 3p strands expressed.

The sequencing data also allowed us to identify precursors on different genomic loci that generate the same mature microRNA. For six mature microRNAs (hsa-let-7f-5p, hsa-miR-16-5p, hsa-miR-181a-5p, hsa-miR-24-3p, hsa-miR-29b-3p and hsa-miR-30c-5p) we were able to identify microRNAs from the other strand derived from the two different precursors. These findings provide evidence that both loci of these particular microRNAs would be expressed in MCF-7 cells. Compared to a similar sequencing based study performed using human umbilical vein endothelial cells (HUVEC) [17], there was an overlap of 80 microRNA sequences.

### Differential expression of microRNAs under hypoxia in MCF-7 cells

**Figure 1:**
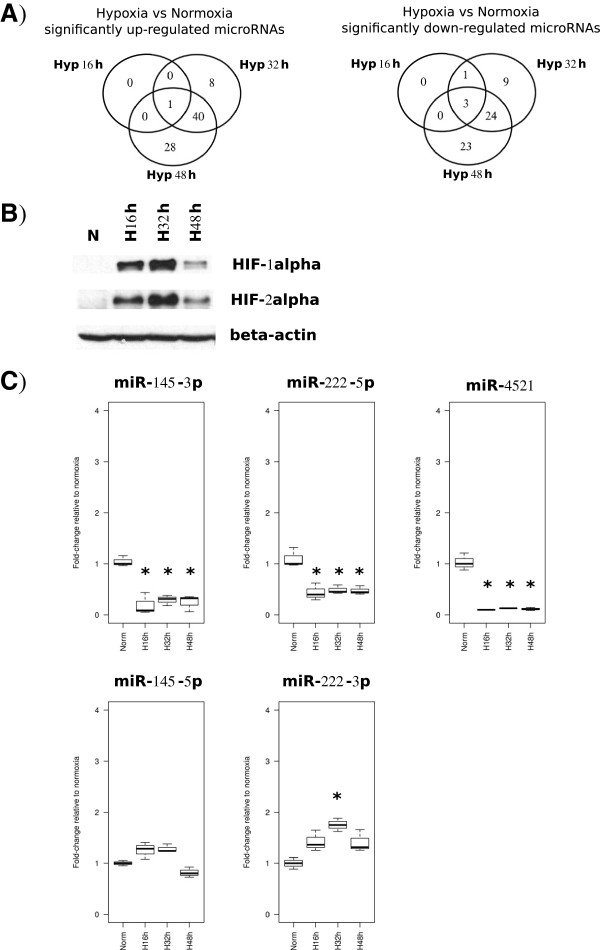
**Hypoxia regulated microRNAs in MCF-7 cells.** microRNA sequencing data generated from a hypoxia time course in MCF-7 (16, 32 and 48 h at 1% Oxygen) was analysed and the overlap between the microRNAs found significantly up- or down-regulated at each time point compared to normoxia control (adj.p-val <0.05) has been represented in Venn diagrams **(A)**. The levels of HIF-1α and HIF-2α were measured by immunoblotting across the hypoxia time course (H16h, H32h, H48h) and normoxia (N), with beta-actin used as a control **(B)**. The down-regulation of miR-4521, miR-145-3p and miR-222-5p in hypoxia was validated by qPCR **(C)**. Fold-changes (in linear scale) obtained for each microRNA at each time point relative to normoxia control are represented in boxplots (*significant fold-change compared to normoxia after ANOVA followed by pairwise t-test, adj.p-val ≤ 0.05). The expression of miR-145-5p and miR-222-3p was also assessed by qPCR, confirming a different pattern of expression as compared to their counterpart strands.

When looking at differential expression between hypoxia and normoxia, we found that the number of differentially expressed microRNAs increased along the time of exposure to hypoxia. We identified 1, 49 and 69 microRNAs up-regulated at 16 h, 32 h and 48 h of hypoxia respectively (adj.p-val ≤ 0.05) whereas 4, 37 and 50 microRNAs were detected as down-regulated at 16 h, 32 h and 48 h of hypoxia respectively (adj.p-val ≤ 0.05) (see Figure 1A). Among each group of significant microRNAs we found microRNAs from both 5p and 3p strands.

All microRNAs significantly changing in expression levels after 16 h of hypoxia remained altered in the same direction across the time course: the up-regulated hsa-miR-210-3p and the down-regulated hsa-miR-4521, hsa-miR-145-3p and hsa-miR-222-5p were also significantly up- and down-regulated, respectively, after 32 h and 48 h of hypoxia whereas the down-regulated hsa-miR-29b-1-5p remained significantly decreased after 32 h of hypoxia but not 48 h (Figure 1A and Tables 1 and 2). The number of significantly up- or down-regulated microRNAs increases substantially at 32 and 48 h of hypoxia, with a considerable overlap between these time points: 40 and 24 microRNAs were found to be significantly up-regulated and down-regulated, respectively, at both hypoxia time points (Figure 1A and Tables 1 and 2, adj.p-val < 0.05). The induction of HIF protein levels across the hypoxia time course was monitored by immunoblotting, showing that both HIF-1α and HIF-2α are induced at 16 h, increase at 32 h of hypoxia and drop at 48 h (see Figure 1B).

**Table 1:** MicroRNAs found significantly up-regulated in MCF7 cells exposed to hypoxia by analysis of microRNA-seq data

**MicroRNAs significantly up-regulated in hypoxia at 16, 32 and 48 h (adj.p-val ≤ 0.05)**						
**microRNAs**	**FC H16h**	**adj.p-val**	**FC H32h**	**adj.p-val**	**FC H48h**	**adj.p-val**
hsa-miR-210-3p	3.24	0.0001	4.03	5.46e-07	3.96	8.41e-07
**MicroRNAs significantly up-regulated in hypoxia at 32 and 48 h (adj.p-val ≤ 0.05)**						
**microRNAs**	**FC H16h**	**adj.p-val**	**FC H32h**	**adj.p-val**	**FC H48h**	**adj.p-val**
**hsa-miR-1**	1.86	0.4806	2.25	0.026223	3.12	0.000682
**hsa-miR-106b-3p**	1.10	0.9629	2.15	0.029336	1.94	0.048003
hsa-miR-1246	1.53	0.9046	5.08	0.000085	3.87	0.001346
**hsa-miR-1269a**	1.18	0.9399	2.43	0.005459	1.94	0.033244
hsa-miR-140-3p	1.11	0.9629	2.21	0.016949	2.01	0.027275
**hsa-miR-141-5p**	1.26	0.9046	1.78	0.049237	1.93	0.018787
hsa-miR-143-3p	1.82	0.2553	2.80	0.000136	1.90	0.021242
hsa-miR-151a-3p	1.35	0.9046	2.47	0.014476	2.34	0.016879
**hsa-miR-181c-3p**	1.12	0.9629	1.89	0.038509	1.84	0.035990
hsa-miR-192-5p	1.33	0.8665	2.14	0.009894	2.24	0.003741
**hsa-miR-194-5p**	1.09	0.9629	1.96	0.042635	2.28	0.008532
**hsa-miR-195-3p**	1.18	0.9555	2.49	0.007142	2.29	0.012846
**hsa-miR-203a**	1.21	0.9242	1.93	0.039759	1.93	0.030635
hsa-miR-215-5p	1.28	0.9046	2.92	0.001669	2.23	0.017671
**hsa-miR-27a-5p**	1.22	0.9046	1.90	0.026536	2.19	0.003759
**hsa-miR-28-3p**	0.97	0.9774	2.15	0.021442	2.04	0.025454
**hsa-miR-3065-3p**	1.27	0.9046	2.13	0.017787	2.67	0.000890
hsa-miR-30d-5p	1.24	0.9046	2.38	0.005459	3.31	0.000050
**hsa-miR-30d-3p**	1.43	0.8010	2.04	0.018042	1.80	0.041199
**hsa-miR-30e-3p**	1.26	0.9046	2.00	0.033309	2.35	0.005147
**hsa-miR-3140-3p**	1.13	0.9629	2.17	0.049237	2.31	0.025454
**hsa-miR-3158-3p**	2.19	0.0594	3.29	0.000032	2.65	0.000730
**hsa-miR-338-5p**	1.15	0.9604	2.11	0.018185	2.72	0.000672
**hsa-miR-33b-5p**	1.27	0.9046	2.87	0.000509	1.97	0.025454
**hsa-miR-203b-3p**	1.18	0.9555	2.11	0.047389	2.26	0.023495
**hsa-miR-3619-3p**	1.30	0.9242	3.11	0.004682	4.15	0.000159
**hsa-miR-3677-3p**	1.77	0.6186	2.94	0.002596	2.73	0.003759
**hsa-miR-378c**	1.02	0.9835	2.11	0.020104	1.88	0.040362
**hsa-miR-378d**	1.04	0.9629	2.39	0.013088	2.08	0.029016
**hsa-miR-378i**	1.38	0.8770	2.63	0.003952	2.69	0.002146
**hsa-miR-3913-5p**	1.06	0.9629	1.91	0.041667	2.26	0.006343
**hsa-miR-3928-3p**	1.22	0.9250	2.28	0.023169	2.12	0.030094
**hsa-miR-4504**	2.14	0.4257	2.97	0.006637	2.55	0.019065
**hsa-miR-4746-5p**	1.37	0.8665	2.13	0.018185	2.17	0.011517
**hsa-miR-4760-5p**	1.82	0.2984	2.04	0.017700	1.80	0.040620
**hsa-miR-548a-3p**	1.12	0.9629	2.55	0.032600	2.33	0.046620
**hsa-miR-627-5p**	1.44	0.8474	2.72	0.002857	2.29	0.011721
**hsa-miR-92b-3p**	1.19	0.9242	1.95	0.032600	2.22	0.006343
**hsa-miR-942-5p**	1.25	0.9046	2.08	0.016836	2.16	0.007682
**hsa-miR-99b-5p**	1.15	0.9629	2.39	0.018617	3.09	0.001097
**MicroRNAs significantly up-regulated in hypoxia by QPCR**^**†**^						
**microRNAs**	**FC H16h**	**adj.p-val**	**FC H32h**	**adj.p-val**	**FC H48h**	**adj.p-val**
**hsa-miR-24-2-5p**	1.08	≥ 0.05	1.65	≤ 0.05	1.81	≤ 0.05
hsa-miR-27a-3p	1.93	≤ 0.05	2.84	≤ 0.05	2.02	≤ 0.05
**hsa-miR-30b-3p**	1.58	≥ 0.05	2.10	≤ 0.05	2.40	≤ 0.05
hsa-miR-30b-5p	1.20	≥ 0.05	2.23	≤ 0.05	1.81	≤ 0.05

MicroRNAs in each section of the table are arranged in numerical order for ease of reading. Fold-change in linear scale (FC) and adjusted p value (adj.p-val) are shown for each microRNA at each hypoxia time. MicroRNAs which regulation by hypoxia has not been reported before are highlighted in bold. Last section shows microRNAs for which sequencing data did not produce significant results but were validated as up-regulated by qPCR (^†^).

Hsa-miR-27a-3p was also found significantly up-regulated across the hypoxia time course in Agilent microarray data.

**Table 2:** MicroRNAs found significantly down-regulated in MCF7 cells exposed to hypoxia by analysis of microRNA-seq data

**MicroRNAs significantly down-regulated in hypoxia at 16,32 and 48 h (adj.p-val ≤ 0.05)**						
**miRNAs**	**FC H16h**	**adj.p-val**	**FC H32h**	**adj.p-val**	**FC H48h**	**adj.p-val**
**hsa-miR-145-3p**	0.16	5.60E-06	0.13	1.23E-07	0.10	1.16E-08
**hsa-miR-222-5p**	0.41	0.02943717	0.34	0.000541974	0.30	7.58E-05
**hsa-miR-4521**	0.16	2.29E-07	0.09	9.76E-12	0.09	7.42E-12
**MicroRNAs significantly down-regulated in hypoxia at 16 and 32 h (adj.p-val ≤ 0.05)**						
**miRNAs**	**FC H16h**	**adj.p-val**	**FC H32h**	**adj.p-val**	**FC H48h**	**adj.p-val**
**hsa-miR-29b-1-5p**	0.41	0.02943717	0.53	0.049237065	0.64	0.160459153
**MicroRNAs significantly down-regulated in hypoxia at 32 and 48 h (adj.p-val ≤ 0.05)**						
**miRNAs**	**FC H16h**	**adj.p-val**	**FC H32h**	**adj.p-val**	**FC H48h**	**adj.p-val**
**hsa-let-7f-1-3p**	0.58	0.618581404	0.37	0.003952372	0.46	0.02280333
**hsa-miR-1260a**	0.59	0.676561681	0.25	0.000224356	0.45	0.027900682
**hsa-miR-1260b**	0.52	0.480646057	0.19	1.21E-05	0.38	0.008394683
hsa-miR-1275	0.98	0.983716794	0.46	0.038509311	0.25	0.000158578
**hsa-miR-15b-3p**	0.92	0.962873516	0.32	0.003952372	0.36	0.007476629
hsa-miR-19a-3p	1.59	0.866491946	0.37	0.036219879	0.37	0.025453723
hsa-miR-19b-3p	1.55	0.866491946	0.33	0.018184509	0.34	0.016879484
**hsa-miR-19b-1-5p**	0.53	0.472759683	0.33	0.002596151	0.31	0.000890148
hsa-miR-22-3p	1.39	0.904640745	0.41	0.039758527	0.40	0.026856552
hsa-miR-221-5p	0.82	0.904640745	0.54	0.041249481	0.36	0.000264743
**hsa-miR-23a-5p**	0.35	0.225229269	0.30	0.016836038	0.16	0.000197073
**hsa-miR-23b-5p**	0.46	0.059350069	0.43	0.003952372	0.43	0.002649597
**hsa-miR-296-3p**	0.46	0.298355571	0.34	0.003952372	0.21	1.55E-05
hsa-miR-32-3p	0.86	0.955522425	0.48	0.036493987	0.33	0.000822847
**hsa-miR-33a-3p**	0.44	0.618581404	0.22	0.012191307	0.17	0.003599729
**hsa-miR-3613-5p**	0.88	0.962873516	0.36	0.002314	0.38	0.002622677
**hsa-miR-424-3p**	0.64	0.720598475	0.31	0.000695205	0.23	9.65E-06
**hsa-miR-4466**	0.53	0.50269118	0.13	1.23E-07	0.19	1.72E-05
**hsa-miR-455-3p**	1.29	0.904640745	0.46	0.026536474	0.45	0.016879484
hsa-miR-505-5p	0.73	0.866491946	0.36	0.001669409	0.35	0.000890148
**hsa-miR-573**	0.66	0.773087527	0.18	1.88E-07	0.09	4.29E-12
**hsa-miR-92a-1-5p**	0.59	0.720598475	0.22	0.000120735	0.20	2.52E-05
**hsa-miR-93-3p**	0.63	0.720598475	0.42	0.016836038	0.37	0.003599729
**hsa-miR-940**	1.11	0.962873516	0.32	0.002314	0.27	0.000255512

MicroRNAs in each section of the table are arranged in numerical order for ease of reading. Fold-change in linear scale (FC) and adjusted p value (adj.p-val) are shown for each microRNA at each hypoxia time. MicroRNAs which regulation by hypoxia has not been reported before have been highlighted in bold.

We have previously demonstrated the up-regulation of hsa-miR-210-3p (previously annotated as hsa-miR-210) across a hypoxia time course in MCF-7 cells [27], but this in depth study has revealed a number of hypoxia regulated microRNAs that have not been reported in previous studies [11-20] (highlighted in bold in Tables 1 and 2).

Agilent microRNA microarrays (3 biological replicates per condition) were also used to complement and validate the sequencing data in a broad manner, as the two technologies have their own strengths and weaknesses in microRNA profiling [28]. We found 228 microRNAs commonly detected between sequencing and microarrays (Additional file 1: Table S3), with a significant correlation between the number of reads and array intensity (Spearman: 0.57 (average), p-val ≤ 0.05). When comparing the fold changes obtained for each microRNA across the time course in each platform there is overall a significant correlation (Pearson: 0.30 (average), p-val ≤ 0.05). Most of the compared microRNAs show a clear trend towards the same direction in both platforms despite some punctual disagreements (see Additional file 2: Figure S1). Moreover around half of the microRNAs found significantly regulated through next-generation sequencing are also found significantly regulated in the microarray experiment (Additional file 2: Figure S1 and Additional file 1: Table S3).

In order to further validate the sequencing results obtained here, we performed qPCR to test the expression levels of hsa-miR-4521, hsa-miR-145-3p and hsa-miR-222-5p. Indeed, all microRNAs were validated as down-regulated in agreement with the sequencing data (Figure 1C, adj.p-val ≤ 0.05). The expression levels of hsa-miR-145-5p and hsa-miR-222-3p were found either not changed or slightly up by qPCR (Figure 1C). This suggests that one strand for these microRNAs is specifically down-regulated under hypoxic conditions and may have been functional as compared to the other strand.

### Role of HIF in microRNA regulation under hypoxia

**Figure 2:**
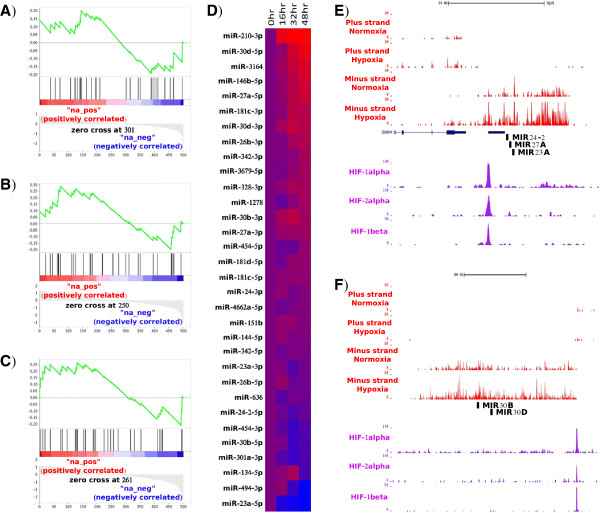
**Hypoxically induced microRNAs are transcriptionally regulated by HIF.** microRNAs within 50 kb of a HIF-binding site were analysed by GSEA using the pan-genomic microRNA datasets of fold change in expression at **(A)** 16 hours, **(B)** 32 hours and **(C)** 48 hours. **(D)** Heat map showing fold change after differing periods of hypoxia for all HIF-binding microRNAs. **(E & F)** RNA-seq and ChIP-seq tracks showing HIF binding and transcriptional up-regulation of previously un-annotated transcripts hosting the loci *MIR27A* and *MIR30D*. Red tracks show strand-specific ribo- RNA-seq in normoxia and hypoxia. RNA-seq reads are split between ones mapping in the plus and minus strand under normoxia or hypoxia. Purple tracks show ChIP-seq using antibodies against the indicated HIF subunit.

Direct regulation of microRNA expression by HIF-1α and HIF-2α in MCF-7 was assessed by ChIP-seq, while stabilization of the two proteins in the same cells under hypoxia has been published previously [25,26]. We used the 500 published high-stringency HIF binding sites [29] in order to look for microRNAs that map to within 50 kb of each binding site. Gene Set Enrichment Analysis (GSEA) against the fold change by hypoxia at each of the three time points shows a weak enrichment for HIF-binding microRNAs amongst those up-regulated by hypoxia, which becomes more apparent at the longer time points (Figure 2A-C). The heat map (Figure 2D) shows the fold change at each time point for each microRNA locus containing a HIF binding site. The most up-regulated microRNA containing a HIF-binding site is indeed hsa-miR-210-3p, but there are clearly other microRNAs (Additional file 1: Table S4). In Figure 2E and F we show the tracks for two loci of particular interest, *MIR30D* and *MIR27A*, which are near the top of the list. Indeed, hsa-miR-30d-5p, hsa-miR-30d-3p and hsa-miR-27a-5p are significantly up-regulated at 32 h and 48 h of hypoxia according to our sequencing data. Both microRNA loci have un-annotated, hypoxia inducible host transcripts that appear in directional ribo-depleted RNA-seq data produced by exposing the MCF-7 cells to hypoxia (24 h 1% hypoxia) [30] and strong HIF-binding ChIP-seq peaks (Figure 2E and F).

**Figure 3:**
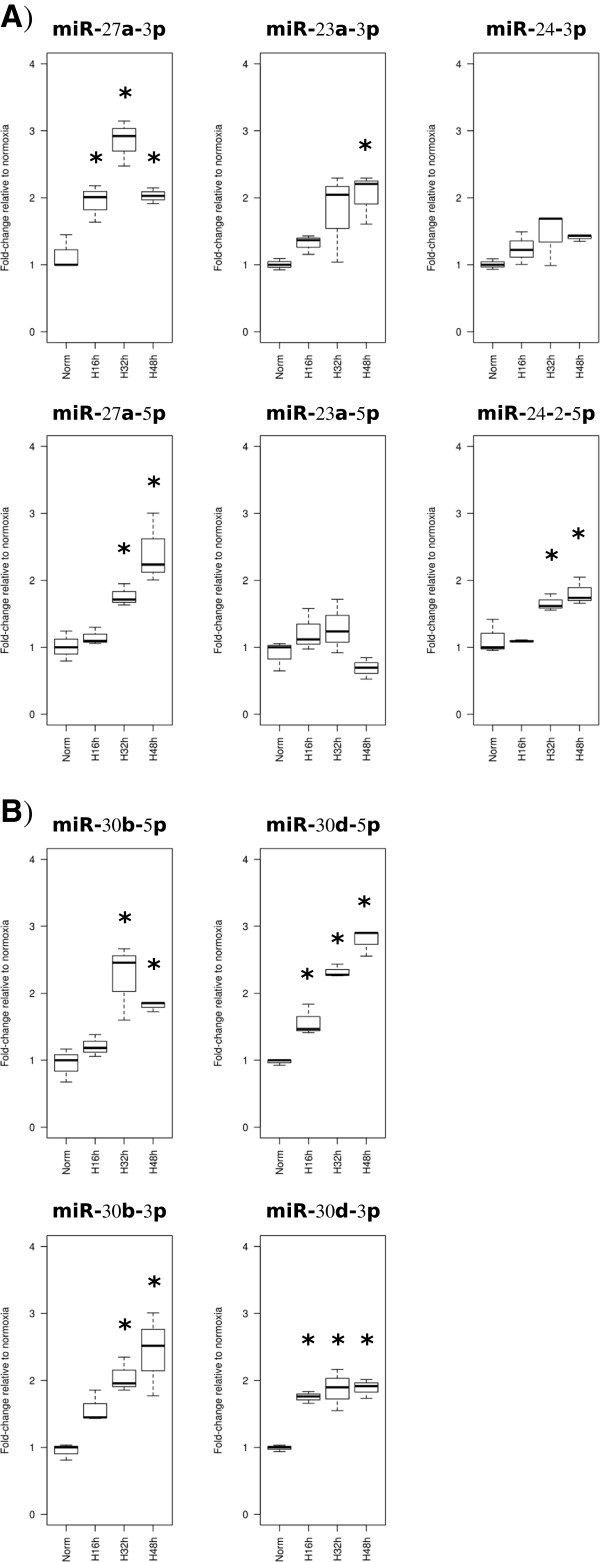
**Hypoxic regulation of microRNAs clustered to *MIR27A *and *MIR30D*.** QPCR was used to assess the expression of both 5p and 3p arms of two microRNA clusters that are within 50 kb of a HIF-binding site: hsa-miR-27a, hsa-miR-23a and hsa-miR-24-2 **(A)** and hsa-miR-30d and hsa-miR-30b **(B)**. Fold-changes (in linear scale) obtained for each microRNA at each time point relative to normoxia control are represented in boxplots (*significant fold-change compared to normoxia after ANOVA followed by pairwise t-test, adj.p-val ≤ 0.05).

Hsa-miR-27a is within a close distance to other two microRNAs, hsa-miR-23a and hsa-miR-24-2, whereas hsa-miR-30d is also very close to hsa-miR-30b. They are reported to be clustered microRNAs that seem to be transcribed in the same pri-miRNA, which agrees with our previous observations concerning the host genes for these microRNAs. Therefore, we checked the expression of these microRNAs by qPCR, validating the up-regulations seen in sequencing results for hsa-miR-30d-5p, hsa-miR-30d-3p and hsa-miR-27a-5p (Figure 3A and B, adj.p-val ≤ 0.05). In addition we found hsa-miR-27a-3p significantly up-regulated across the whole hypoxia time course, hsa-miR-23a-3p up-regulated at 48 h and hsa-miR-24-2-5p, hsa-miR-30b-5p and hsa-miR-30b-3p significantly up-regulated at 32 and 48 h (Figure 3A and B, adj-p-val ≤ 0.05). It is worth to mention that hsa-miR-27a-3p was also found significantly up-regulated across all hypoxia time course in Agilent microarray data (Additional file 1: Table S3). All these results would further support the role of HIF on regulating the expression of these microRNA clusters under hypoxic conditions. The results link genome wide HIF occupancy under hypoxic conditions to the up-regulation of pri-miRNAs and mature microRNAs.

### Association of hypoxia regulated microRNAs with a hypoxia gene signature in breast cancer samples

Previously, we have reported the direct correlation of hsa-miR-210-3p with a hypoxia score based on the expression of 99 genes [31] in a series of breast cancer tumours [27]. Since microRNA expression profiles for this breast cancer series have been recently published (GSE22220) [32], we have investigated the correlation of the hypoxia score with microRNA expression in this series. Among the positively correlated microRNAs to hypoxia in breast cancer (Spearman rank test, adj.p-val ≤ 0.05), there are two microRNAs that overlap with our list of hypoxia induced microRNAs: hsa-miR-210-3p and hsa-miR-27a-3p, which is derived from the *MIR27A* cluster (see Table 3). Interestingly another microRNA generated from the same cluster, hsa-miR-24-3p, is also directly correlated to hypoxia in breast cancer (Table 3). Although no regulation by hypoxia has been detected for this microRNA in our MCF-7 cells, this could be due to the fact that hsa-miR-24-3p is encoded in two different genomic regions and therefore can be subjected to other regulatory mechanisms that contribute to its final levels. However it is important to highlight that three of the microRNAs for which we have identified a HIF binding site at a close distance appear to be directly correlated to hypoxia in breast cancer. This data demonstrates for the first time that hsa-miR-27a-3p and hsa-miR-24-3p are regulated by hypoxia *in vivo* in breast cancer and that this regulation is possibly dependent on HIF.

**Table 3:** MicroRNAs found up-regulated in hypoxic MCF-7 and positively correlated to hypoxia in breast cancer

**miRNAs**	**rho**	**adj.p-val**
**hsa-miR-210-3p**	0.52	1.12E-15
**hsa-miR-27a-3p**	0.31	5.03E-06
**hsa-miR-24-3p**^†^	0.36	1.15E-07

MicroRNAs are arranged according to significance to hypoxia signature in breast cancer. Correlation coefficient (rho) and adjusted p value (adj.p-val) are shown.

^†^Not significantly regulated in hypoxia in MCF-7 cells. However it is part with hsa-miR-27a-3p of the same cluster, which has a HIF binding site mapped close.

### microRNAs encoded within host genes

**Figure 4:**
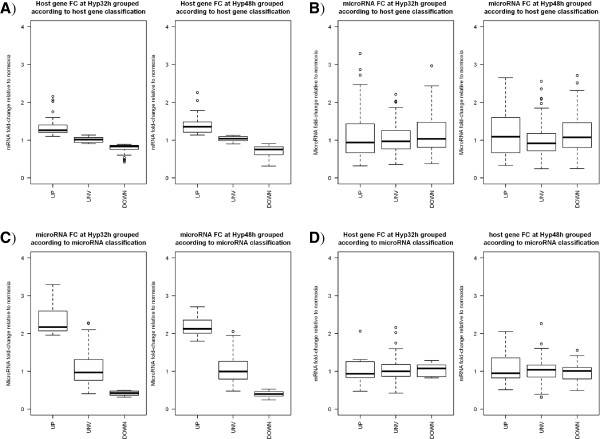
**Low correlation between microRNA and corresponding host gene expression.** Expression of genes hosting microRNAs was obtained from microarray data and expression of corresponding microRNAs was obtained from microRNA-seq data. The hypoxic regulation at 32 h and 48 h for both groups was then compared from two different perspectives. First, hosting genes were sorted in 3 groups at each time point: significantly down-regulated (down), not significantly regulated (unv) and significantly up-regulated (up). The fold-change distribution for each group of genes at 32 h and 48 h of hypoxia compared to normoxia is shown in boxplots **(A)**. For each group of genes, the fold-change distribution of corresponding microRNAs at 32 h and 48 h compared to normoxia is also shown in boxplots for comparison **(B)**. Second, microRNAs hosted within genes were sorted in 3 groups according to their hypoxic regulation at 32 h and 48 h: significantly down-regulated (down), not significantly regulated (unv) and significantly up-regulated (up). The fold-change distribution for each group of microRNAs at 32 h and 48 h of hypoxia compared to normoxia is shown in boxplots **(C)**. For each group of microRNAs, the fold-change distribution of corresponding host genes at 32 h and 48 h compared to normoxia is also shown in boxplots for comparison **(D)**. All fold-change distributions are shown in linear scale.

**Figure 5:**
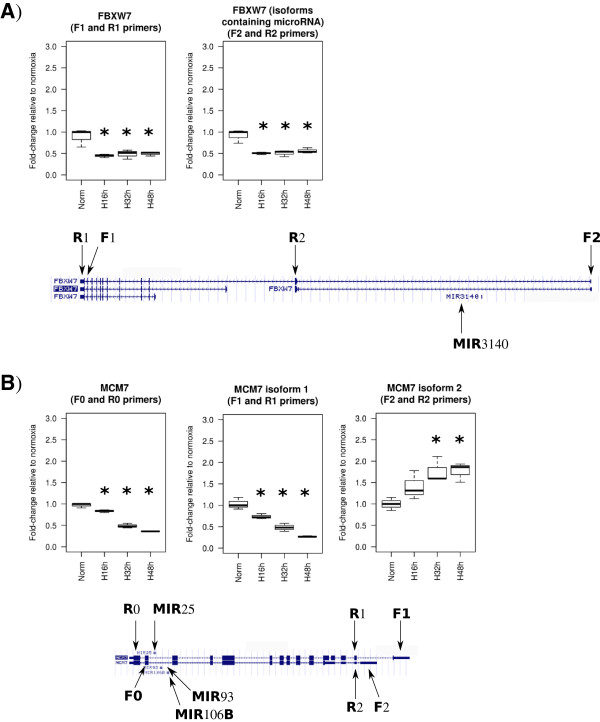
**Examples of lack of correlation between microRNA and host gene expression.** QPCR was used to assess the expression of two genes hosting microRNAs: *FBXW7***(A)** and *MCM7***(B)**. Primers able to anneal to different isoforms were used in order to monitor isoform-specific expression. Fold-changes (in linear scale) obtained for each transcript at each hypoxia time point relative to normoxia control are represented in boxplots (*significant fold-change compared to normoxia after ANOVA followed by pairwise t-test, adj.p-val ≤ 0.05). For *FBXW7* and *MCM7*, diagrams showing the different isoforms and the location of microRNAs and pairs of primers designed for detecting each isoform are included in **(A)** and **(B)**, respectively (forward and reverse primers are designed as F and R, respectively).

Some of the microRNAs found up- or down-regulated under hypoxia are located within intronic regions of protein coding genes. Therefore, we generated mRNA expression profiles of the same time course samples using Illumina arrays. If only considering microRNAs with a unique genomic position, we found 185 microRNAs encoded within 120 host genes that were detected in our microarray experiment (Additional file 1: Table S5). No correlation was found between microRNA expression and host gene expression at any hypoxia time point (Spearman rank correlation: 0.137 (P.val = 0.0613) at 16 h; -0.0949 (P.val = 0.1961) at 32 h; -0.0127 (P.val = 0.8621) at 48 h). Looking at the expression of genes containing microRNAs, we observed that they do not show a lot of variation. When dividing them in 3 groups according to whether they were significantly up-regulated, significantly down-regulated or without changes in hypoxia, the average fold-change of the up- or down-regulated groups is not very different to the group without changes (Figure 4A), suggesting that these genes are very lightly regulated by hypoxia at transcriptional level. For each of the group of genes, we represented boxplots with the expression of the microRNAs they contained (Figure 4B), showing the lack of correlation between the average fold-change of the microRNAs and the average fold-change of the corresponding host genes. Similarly from the microRNA point of view, we divided the microRNAs located within a host gene in three groups depending on their fold-change expression at 32 h and 48 h of hypoxia (up-regulated, down-regulated or no change, Figure 4C). Then for each group of microRNAs, we represented the fold-changes of the corresponding host genes (Figure 4D). While up-regulated and down-regulated microRNAs showed an average fold-change of 2 and 0.5 respectively (Figure 4C), the average fold-change of the corresponding host genes was very similar and close to 1 for both groups (Figure 4D). The picture is the same when Agilent microarray data are used to determine microRNA expression fold changes instead of small RNA-seq (Additional file 3: Figure S2). Detailed examples of these relationships can be seen in Additional file 1: Table S6. In general we observed around a 50% agreement between microRNA and host gene expression. In order to gain more information, we focused on cases where there is no correlation between microRNA and host gene expression and performed additional validations. The first example is hsa-miR-942-5p, found significantly up-regulated at 32 and 48 h, whereas its hosting gene *TTF2* was significantly down-regulated in array data (Additional file 1: Tables S5 and S6). Indeed *TTF2* was found significantly down-regulated in hypoxia by qPCR (adj.p-val ≤ 0.05, data not shown). Also hsa-miR-3140-3p is up-regulated in sequencing data whereas the host gene *FBXW7* is down-regulated in array data (Additional file 1: Tables S5 and S6). *FBXW7* has four isoforms and only two would contain *MIR3140* within its sequence (Figure 5A). We designed a pair of primers (Figure 5A, F1 and R1) that would amplify three isoforms, including two that do not include the *MIR3140 locus* and a pair of primers specific for the two isoforms containing *MIR3140* (Figure 5A, F2 and R2). All products were found significantly down-regulated in hypoxia by qPCR (Figure 5A, adj.p-val ≤ 0.05).

Another interesting example is the cluster composed by hsa-miR-106b and hsa-miR-93 and their host gene *MCM7*. The microRNA sequencing data showed a significant up-regulation of hsa-miR-106b-3p and down-regulation of hsa-miR-93-3p at 32 h and 48 h of hypoxia, whereas *MCM7* was significantly down-regulated according to array data (Additional file 1: Tables S5 and S6). *MCM7* has two isoforms and was confirmed as significantly down-regulated in hypoxia by qPCR when primers able to amplify both isoforms were used (Figure 5B, primers F0 and R0). However when using specific primers for testing the expression of each isoform separately, we found one isoform significantly down-regulated at all hypoxia times (Figure 5B, primers F1 and R1, adj.p-val ≤ 0.05) whereas the other isoform was significantly up-regulated at 32 and 48 h (Figure 5B, primers F2 and R2, adj.p-val ≤ 0.05). This isoform specific regulation would match the regulation of the microRNAs provided they are processed from preferred isoforms.

### Changes in expression of microRNA processing genes due to hypoxia

**Figure 6:**
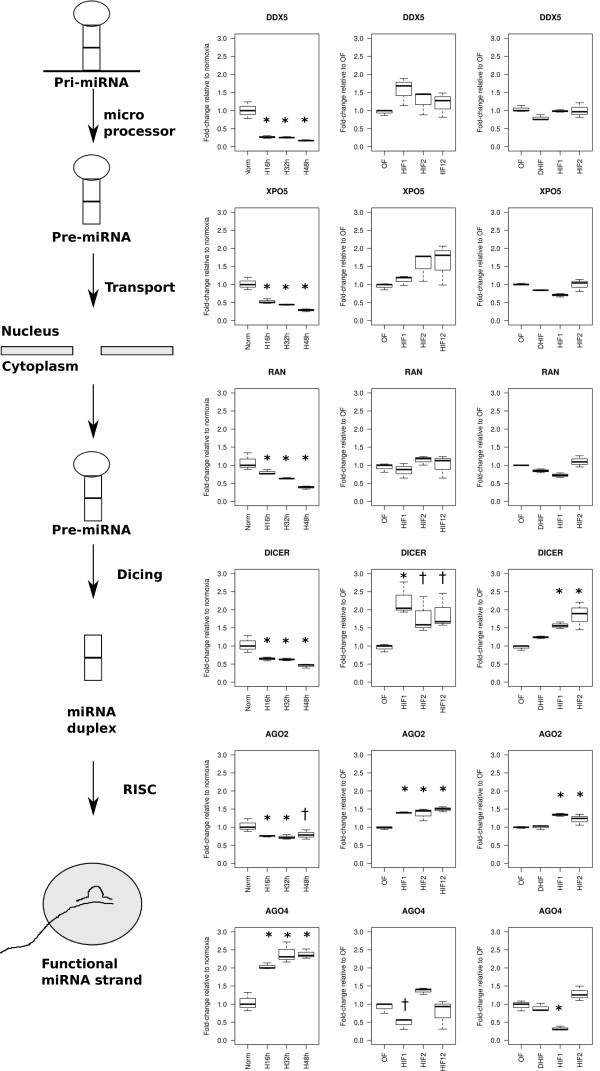
**Hypoxia regulates genes involved in microRNA processing pathway in a HIF-dependent manner.** QPCR was performed to monitor the expression of genes involved in the microRNA processing pathway. Hypoxia MCF-7 time course and 2 sets of HIF-1α and HIF-2α siRNA in MCF-7 were analysed. Fold-changes (in linear scale) obtained for each gene at each time point relative to corresponding control are represented in boxplots (*significant fold-change compared to normoxia after ANOVA followed by pairwise t-test, adj.p-val ≤ 0.05; † adj.p-val ≤0.08).

Overall there is a certain degree of coordination between host gene and microRNA expression under hypoxia but also discrepancy. This suggests that other factors may be involved in the changes observed in mature microRNA expression under hypoxia. We used the gene expression profiles to specifically monitor any change in the expression of genes coding for proteins involved in the microRNA maturation pathway in hypoxic MCF-7 cells. Indeed, significant changes (see Additional file 1: Table S7, adj.p-val < 0.05) were detected in the expression of several genes, including *DDX5* (encoding P68 which forms part of the protein complex lead by DROSHA), *XPO5* and *RAN* (encoding EXPORTIN5 and RAN, respectively, both involved in microRNA transport from the nucleus to the cytoplasm), *DICER* and *EIF2C2* and *EIF2C4* (encoding AGO2 and AGO4, respectively, both associated to RISC complex). We also found that some of these changes were HIF-1α and HIF-2α dependent based on previous data, in which gene expression regulations dependent on these transcription factors were studied through microarray profiles after short interfering (si) RNA-based suppression of HIF subunits [26]. In order to validate these results, we performed qPCR using the MCF-7 hypoxia time course, and two HIF-1α and HIF-2α siRNA sample sets (Figure 6). Indeed, we could confirm a significant down-regulation of *DDX5*, *XPO5*, *RAN*, *EIF2C2* and *DICER* consistently across the hypoxia time course in MCF-7 cells, as well as a significant up-regulation of *EIF2C4* (Figure 6). We also confirmed by qPCR a significant up-regulation of both genes *DICER* and *EIF2C2* when HIF-1α and HIF-2α are silenced in MCF-7 compared to the control, in both independent sample sets (Figure 6). Concerning *EIF2C4*, its expression was found significantly down-regulated upon HIF-1α silencing (Figure 6). Therefore the down-regulation of *DICER* and *EIF2C2* under hypoxia is dependent on both HIF-1α and HIF-2α, whereas the up-regulation of *EIF2C4* seems to be only dependent on HIF-1α. Changes observed in *DDX5*, *XPO5* and *RAN* in hypoxic MCF-7 seem to be independent of HIF-1α or HIF-2α, as their expression did not change in the HIF siRNA experiments (Figure 6).

Despite the fact that some of the changes observed under hypoxia for these genes are HIF dependent, we could not find any high stringency HIF binding site close to any of these genes in our datasets. Since the down-regulation of *DICER* in hypoxic MCF-7 cells is the most strongly associated to HIF among the genes involved in microRNA processing, we decided to test whether this change was also observed *in vivo*. We used the gene expression data generated for the same breast cancer series we have referred in the previous section (GSE22220) [32] and investigated the correlation of *DICER* expression with the hypoxia score based on the expression of 99 genes (univariate analysis) [31]. Interestingly, we found that the expression of *DICER* was significantly inverse correlated to hypoxia in breast cancer (univariate analysis: rho = -0.37, pval = 3.e-08), meaning that the expression of *DICER* is lower as hypoxia increases. Therefore we show that *DICER* is also down-regulated under hypoxia *in vivo* in breast cancer and that this regulation is possibly dependent on HIF although not in a direct manner.

## Discussion

We have presented a very robustly analysed, deep sequencing based microRNA expression profile of a three point hypoxia time course, representing a first for MCF-7 cells. To our knowledge, this is the first time such an in-depth study has been performed and all the data is publicly available at GEO (reference series: GSE47534) as a resource for future investigations. We have shown that the number of hypoxia regulated microRNAs increases in time, with a clear breakpoint between 16 h and 32 h. This is in agreement with Kulshreshtha and colleagues [16], although spotted microarrays provided a limitation of their study. Overall, 69 microRNAs were found regulated in more than one time point, of which 56 are novel for hypoxia. In particular, we report here the down-regulation of hsa-miR-4521, hsa-miR-145-3p and hsa-miR-222-5p at 16 h, 32 h and 48 h of hypoxia. These changes were detected along the whole time course in addition to the up-regulation of hsa-miR-210-3p. Therefore, these as yet largely uncharacterised microRNAs could play an important role in hypoxia. Since hsa-miR-210-3p has proved to be a master regulator (for a review see [33]) and an independent prognostic factor in breast cancer [27], it will be worth to assess these microRNAs for their relevance as breast cancer prognostic markers in the future. A top predicted target of hsa-miR-145-3p in miRBase (DIANA MICRO -T and MIRDB) is *CCNC* (cyclin C), a possible tumor suppressor gene [34], while a top predicted target for hsa-miR-4521 is *FOXM1* (miRBase, using DIANA MICRO -T and MIRDB), which controls cell cycle gene expression [35]. It is therefore conceivable that the down-regulation of these microRNAs does influence cell proliferation.

In addition, we report other microRNAs that are consistently regulated at 32 h and 48 h in MCF-7 cells. Some of these changes have been reported before, using other cell types and conditions, such as the up-regulation of hsa-miR-151-3p in nasopharyngeal carcinoma cells [15], hsa-miR-30d-5p in both nasopharyngeal carcinoma cells and HT29 colon cancer cells [12,15], hsa-miR-140-3p and hsa-miR-143-3p in human primary pulmonary artery smooth muscle cells (PASMCs) [18], hsa-miR-27a-3p and hsa-192-5p in colon and breast cancer cells and PASMCs [16,18], and hsa-miR-1246 and hsa-miR-215-5p in colonic epithelial cells [20]. Also the down-regulation of hsa-miR-32-3p and hsa-miR-505-5p has been reported in HUVEC cells [14]. In general, the overlap between studies is limited due to the usage of different cell lines and hypoxia conditions, as well as to the variety of platforms used for the measurement of microRNA expression. It is also not uncommon to find microRNAs for which contrasting regulation under hypoxia has been described. To support this, an extensive comparison of our data at each hypoxia time point with previous studies [11-16,18-20] has been included in Additional file 1: Table S9.

It is known that prolonged hypoxia leads to generation of free radicals and therefore to oxidative stress. Some of the microRNAs found to be regulated by hypoxia in this study have been reported in the context of oxidative stress. For instance, hsa-miR-210-3p regulates mitochondrial activity under hypoxia by targeting *ISCU1*, *ISCU2*, *COX10*, *SDHD* and *NDUFA4* and also affects the production of Reactive Oxygen Species (ROS). Indeed it has been found that hypoxia increases ROS levels in cancer cells and that this effect can be reversed by blocking miR-210-3p. However ROS levels are not altered by hypoxia in endothelial cells but they are increased upon hsa-miR-210-3p abrogation. In other cells such as differentiated skeletal muscle cells, neonatal rat cardiomyocytes and H9c2 cells, miR-210-3p expression reduces mitochondrial ROS production and decreases mitochondrial mass (all reviewed in [36]). Therefore further work is needed to understand the role of this microRNA in the regulation of ROS levels. It has been also reported that the treatment of auditory cells with the potent oxidant t-BHP down-regulates the expression of hsa-miR-203a and hsa-miR-28-3p (which we found up-regulated in hypoxia at 32 h and 48 h, Table 1) and hsa-miR-92a-1-5p (which we found down-regulated at 32 h and 48 h of hypoxia, Table 2) [37]. Furthermore, the expression of the hypoxia regulated hsa-miR-143-3p, hsa-miR-27a-3p (Table 1) and hsa-miR-22-3p (Table 2) has been described to be altered in human fibroblasts after treatment with peroxide and etoposide (only hsa-miR-27a-3p) [38].

The next question to address was the role of HIF in this process as a key mediator of the transcriptional response to hypoxia. In previous work [29,39], it was demonstrated that HIF-1α and HIF-2α binding to DNA is associated with up-regulation of nearby genes, but the association of binding sites and microRNA expression at whole genome level had not been explored so far. Only two microRNAs, hsa-miR-26a and hsa-miR-210, have been shown to have closely positioned HIF binding sites by HIF-1α chromatin immunoprecipitation (ChIP) [16]. Here, we used HIF-1α and HIF-2α ChIP-seq data generated from MCF-7 cells to locate HIF binding sites close to microRNAs at genome-wide level. GSEA using up- and down-regulated microRNAs revealed an association between proximity of HIF binding and up-regulation. This is further supported when comparing our data to previous studies, since HIF binding sites identified here are closer to microRNAs reported to be up-regulated rather than down-regulated in hypoxia (Additional file 1: Table S9). We showed that functionally important, up-regulated microRNAs have HIF binding sites, such as hsa-miR-27a and hsa-miR-30d. Both microRNAs are involved in cancer progression, hsa-miR-30d-5p by targeting TP53 [40] and hsa-miR-27a-3p by regulating a number of transcription factors such as ZBTB10 [41,42]. The HIF dependence of the microRNA cluster composed by hsa-miR-27a, hsa-miR-23a and hsa-miR-24-2 had been discussed earlier through the prediction of a conserved HIF binding site within the promoter region of this microRNA cluster [43]. In addition to the HIF binding site, we show here that the expression of hsa-miR-27a-3p and hsa-miR-24-3p is positively correlated with a hypoxia score based on the expression of 99 genes in a series of breast cancer samples. This would reinforce the role of HIF in regulating microRNAs in breast cancer and other solid tumours, and the idea that hypoxia could be a key factor in microRNA modulation in cancer [16].

Nevertheless, not all the microRNAs with HIF binding sites in their proximity were up-regulated under hypoxia. We also found some discrepancies between microRNA and host gene expression (where microRNAs are located within intragenic regions), and have shown in the example of the *MCM7* locus, that differential expression of transcript isoforms may play a role in these discrepancies. In general, host gene expression was less responsive to hypoxia than changes in levels of mature microRNA forms. This is in agreement with findings that other levels of microRNA regulation take place. Indeed, it has been shown that hypoxia is able to regulate the expression and activity of proteins involved in the post-transcriptional microRNA processing. In HUVEC cells exposed to chronic hypoxia (24 h), several components of the processing pathway as DGCR8, EXPORTIN5, DICER, TRBP, AGO1 and AGO2 are significantly down-regulated at both protein and mRNA level [14]. Moreover, the decrease of DICER protein and mRNA levels has been confirmed *in vivo* in several tissues from mice exposed to chronic hypoxia and also low mRNA levels for DICER have been observed in rat models for pulmonary arterial hypertension [44]. On the other hand, it has been shown in PASMCs that AGO2 can be subjected to a post-translational modification that increases its stability and translocation rate to the stress granules. Since AGO2 is the key mediator of microRNA function, this modification of AGO2 has an impact on microRNA – target interaction [18]. Some of our findings are in agreement with these recent reports, such as the down-regulation of XPO5, DICER and AGO2 transcripts. In addition, we found a significant down-regulation of *DDX5* and *RAN*, and a significant increase in AGO4 transcript. We have further explored the regulation of these genes under hypoxia in other 13 cell lines by using expression data available in GEO from other hypoxia studies (see Additional file 1: Table S10). These cell lines were exposed to hypoxia (mostly 1% Oxygen) for a period ranging between 16 h and 72 h. In general we can see that *DICER*, *DDX5*, *XPO5* and *RAN* are down-regulated at transcriptional level in several cell lines but not all, suggesting that these changes have a component of cell specificity (Additional file 1: Table S10). *EIF2C2* (AGO2) transcript presents contrasting regulation depending on the cell line whereas *EIF2C4* (AGO4) appears to be up-regulated in two other cell lines (HUVEC and prostate cancer at 48 h and 72 h of hypoxia, respectively. Additional file 1: Table S10). Although the transcript levels do not always correspond to protein levels, previous studies in hypoxia (as we have just referred above) show a good concordance between both for several of the microRNA processing genes. DDX5 acts also as a transcription cofactor and is required for cell proliferation [45], for induction of TP53 dependent p21 (CDKN1A) expression after DNA damage [46] and NOTCH1 signalling [47]. The effect of AGO4 on microRNA levels is not clear, but it could provide a further enhancement of RISC complex mediated effects on translation. Despite the fact that several members of the processing machinery are down-regulated, recent work has shown that hydroxylation [18] and phosphorylation [24] of AGO2 affect the microRNA maturation process.

The role of HIF in the regulation of the genes involved in the microRNA processing has not been thoroughly studied. Using HIF siRNA on MCF-7 cells, we showed that indeed DICER and AGO2 transcriptional down-regulations in hypoxia are HIF-1α and HIF-2α dependent, while this dependency was not observed in HUVEC cells [14]. Also, AGO4 transcriptional up-regulation in hypoxia is HIF-1α dependent. Since no HIF binding site was mapped close to these genes in our datasets, the regulation is likely to be indirect and in line with the observation that in breast cancer samples low levels of DICER are associated with a hypoxia signature.

HIF is an important factor in the pathophysiology of cancer and the identification of the mechanisms through which it mediates microRNA expression regulation will lead to a better understanding of this complex pathway. In addition, establishing a complete repertoire of hypoxia regulated microRNAs will lead to more detailed analysis of large scale cancer sample datasets for microRNA-target gene relationships [48] and lead to the development of advanced prognostic assays and therapeutic approaches.

## Conclusions

Next generation sequencing based methods are state of the art for microRNA profiling. Here we applied this methodology and identified a high number of microRNAs that are regulated by hypoxia in MCF-7 cells, including novel hypoxia regulated microRNAs detected in this study that are candidates for developing breast cancer prognostic assays. The microRNA data were integrated with mRNA and HIF ChIP-seq data to show genome wide correlation between HIF binding and microRNA regulation for the first time. In addition, we show that hsa-miR-27a-3p and hsa-miR-24-3p, for which we have identified a HIF binding site at close distance, are positively correlated to hypoxia in breast cancer. Analysis of microRNA-host gene expression correlation supports the idea that posttranscriptional mechanisms for microRNA expression regulation are in action under hypoxia, and we provide evidence that HIF can play a role by modulating the expression levels of microRNA processing genes such as the down-regulation of DICER. Moreover we report an inverse correlation of *DICER* expression to hypoxia in breast cancer. Therefore, HIF, as a key factor of the hypoxia transcriptional response plays a role in the regulation of microRNAs either directly through binding to microRNA loci, or indirectly through regulating microRNA processing gene expression and pathways leading to posttranslational modifications of AGO2.

## Methods

### Cell culture and hypoxia exposure

Breast adenocarcinoma cell line MCF-7 cells were grown in Dulbecco’s Modified Eagle Medium supplemented with 10% fetal bovine serum (Sigma-Aldrich, St Loius, Missouri, USA) and 2 mmol/L of L-glutamine. Exposure of cell cultures to hypoxia (1% Oxygen) was undertaken in an In vivo2 Hypoxia Work Station (Ruskin Technologies, Pencoed, UK) in parallel with cells maintained in normoxic conditions (21% oxygen). Cells were recovered after 16 h, 32 h and 48 h of exposure to hypoxia. All experiments were done in triplicate from independent cell cultures.

### RNA extraction

RNA was extracted from cells using the miRVana miRNA Isolation Kit (Ambion, Life Technologies, Carlsbad, California, USA). RNA quality and abundance were determined after extraction using an Agilent 2100 Bioanalyzer (Agilent Technologies, Santa Clara, California, USA) and a Nanodrop ND-1000 spectrophotometer (Thermo Fisher Scientific, Waltham, Massachusetts, USA), respectively.

### Preparation of microRNA libraries for next-generation sequencing

For each experimental condition (normoxia, 16 h hypoxia, 32 h hypoxia, 48 h hypoxia), small RNA libraries for next-generation sequencing were prepared from RNA samples obtained from two biological replicates. Each library was prepared from the 20 to 30 nucleotide RNA fraction. This was isolated from 2 μg of total RNA after being run in a 15% urea-TBE gel (Invitrogen, Life technologies, Carlsbad, California, USA) for 1 h at 200 V. The RNA contained in the excised band was eluted in 300 μl of 0.3 M NaCl by 4 h of constant rotation at room temperature. The elute was separated from the gel debris using a Spin-X-column (Thermo Fisher Scientific, Waltham, Massachusetts, USA) and RNA was precipitated by adding 750 μl of 100% ethanol and 3 μl of glycogen (1 mg/ml) and incubating for 30 minutes at -80°C. The precipitated RNA was centrifuged at 14,000 rpm for 25 minutes at 4°C, washed with 75% ethanol, air dried and resuspended in 5 μl of RNAse-free water. This material was used to prepare sequencing libraries using the DGE-small RNA sample preparation kit (v1.5) from Illumina (Illumina inc., San Diego, CA, USA ) according to the protocol provided by the manufacturer. The abundance of the libraries was measured using Qubit dsDNA HS Assay kit and a Qubit 2.0 fluorometer (both from Life technologies, Carlsbad, California, USA). The size of the products contained in the libraries was assessed with a DNA 1000 chip and an Agilent 2100 Bioanalyser (both from Agilent Technologies, Santa Clara, California, USA). The libraries were sequenced using an Illumina Hiseq sequencer according to the manufacture’s standard protocols at 36 bp read length.

### Alignment and analysis of microRNA next-generation sequencing data

Small RNA sequencing data were processed from raw FASTQ files and analysed using the Kraken pipeline (http://www.ebi.ac.uk/research/enright/software/kraken) [49]. The 3’ adaptors were stripped using the criteria: 12-nt alignment stretch with no more than 2 mismatches and no gaps. The reads were then filtered for low-complexity regions and the remaining reads were size-selected for 18-26nt, resulting in inclusion of more than 75% of reads on average for each sample (Additional file 1: Table S1). A file with unique reads and their corresponding counts was generated for each sample. The final processed unique reads were mapped to the human genome (hg19 based on Ensembl v64 [50]) using Bowtie 0.12.7 [51] allowing for two mismatches and using the best alignment stratum option. Reads mapped to more than 20 loci were discarded. Mapped reads were classified and counted based on genomic annotations (RNAs, coding genes, pseudogenes and repeats) obtained from Ensembl v64 (Additional file 1: Table S1). For reads mapping to multiple loci, counts were divided by total number of reads. The sense and antisense distribution of mapped reads across all chromosomes was also analysed.

For differential expression of microRNAs, the overlap between mapped reads and human mature microRNAs (based on miRBase v17 [52]) was found using the function *findOverlaps* available in the Bioconductor GenomicRanges package (http://bioconductor.org/packages/2.6/bioc/html/GenomicRanges.html). The normalization and differential expression was performed with Bioconductor edgeR package v2.3.57 [53] using both common and tagwise dispersion. The significant differentially expressed microRNAs were determined by an adjusted p value lower than 0.05 based on the Benjamini and Hochberg multiple testing correction [54]. Data has been submitted to GEO (reference series: GSE47534; miRNA-seq: GSE47602).

### HIF-1α and HIF-2α ChIP-seq analysis

We used the 500 published high-stringency HIF binding sites [29] in order to look for microRNAs that map to within 50 kb of each binding site. Gene Set Enrichment Analysis (GSEA) was carried out as described previously [29].

### microRNA microarrays

microRNA expression profiles were performed using the human microRNA microarray V2 (Agilent Technologies, Santa Clara, California, USA) which contains 723 human microRNAs (Sanger database V.10.1) and 76 human viral microRNAs. Three biological replicate samples for each experimental condition (normoxia, 16 h hypoxia, 32 h hypoxia and 48 h hypoxia) were analysed. Samples were prepared with the Mirna Labelling Reagent and Hybridization kit (Agilent Technologies, Santa Clara, California, USA) following the instructions supplied by the manufacturer. The microarray hybridization was performed at 55°C for 20 h and at a constant speed rotation of 20 rpm. The hybridization signals were detected by the Agilent Microarray scanner G2565BA and quantified using the Agilent Feature extraction software version 9.5.1 (both from Agilent Technologies, Santa Clara, California, USA). A GeneView file including the Total Gene Signal (raw data) and the Total Gene Error for each of the microRNAs interrogated on the microarray was generated for all the samples and used for further analysis. One sample corresponding to MCF-7 cells exposed to hypoxia for 16 h was affected by a random technical problem and had to be removed due to the bad quality of the scan.

### microRNA microarray data analysis

The Total Gene Signal was normalised using a variance stabilization method described by Huber and colleagues [55]. Furthermore a detection factor was calculated for each microRNA in every sample as the following subtraction: Total Gene Signal - Total Gene Error. A microRNA was considered detected when the value of this factor was higher than zero. The normalised data was then filtered based on the detection factor and on the microRNA class. The minimal requirements for a microRNA to be selected for further analysis were: (i) detection in at least two biological replicates of any experimental condition and (ii) be included as human microRNA in miRBase (http://www.mirbase.org). Limma analysis was then performed to assess independently the effects of 16 h, 32 h and 48 h of hypoxia exposure compared to normoxic control in microRNA expression levels in MCF-7 cells. Benjamini and Hochberg method was used to correct for multiple testing [54]. microRNAs with adjusted p values lower than 0.05 were considered significantly regulated. Data has been submitted to GEO (reference series: GSE47534; miRNA: GSE47532).

### Gene expression microarrays

mRNA expression was measured using Illumina HumanWG-6 v3.0 Expression Bead Chip arrays (Illumina inc., San Diego, CA, USA). The Illumina platform was chosen as it performed well in large comparative studies [56]. Three biological replicate samples for each experimental condition (normoxia, 16 h hypoxia, 32 h hypoxia and 48 h hypoxia) were analysed. RNA was amplified using the Illumina Total Prep RNA Amplification Kit (Ambion, Life Technologies, Carlsbad, California, USA). Amplified RNA product (850 ng) was hybridised to the Illumina microarrays using single chamber hybridization cartridges. Washing, staining and scanning were carried out as specified in the Illumina Whole Genome Expression Manual v.1.

### Gene expression microarray data analysis

Average signal was background subtracted with local background subtraction (BeadStudio) and consolidated per gene. Data was quantile normalized in Bioconductor (http://www.bioconductor.org) and filtered based on gene detection level using the pvalDet parameter. Only genes with pvalDet lower than 0.05 in at least 2 replicates of any of the experimental condition were considered for further analysis. Limma analysis was then performed to assess independently the effects of 16 h, 32 h and 48 h of hypoxia exposure compared to normoxic control in gene expression levels in MCF-7 cells. Benjamini and Hochberg method was used to correct for multiple testing [54]. Genes with adjusted p values lower than 0.05 were considered significantly regulated. Data has been submitted to GEO (reference series: GSE47534; mRNA: GSE47533).

Datasets from published studies (presented in Additional file 1: Table S10) have been analysed as described above.

### Real time PCR (qPCR) analysis

MicroRNA expression was assessed by qPCR with TaqMan microRNA assay protocol (Applied Biosystems, Life Technologies, Carlsbad, California, USA) using 5 ng of total RNA per microRNA as indicated by the manufacturer. QPCR was done in a CFX96 real-time PCR detection system (BioRad, Hercules, California, US). Cycling conditions included a pre-incubation step (95°C for 3 minutes) and 40 cycles of amplification (95°C for 30 seconds, 60°C for 1 minute). For gene expression, total RNA (2 μg) was reverse transcribed using the SuperScript II Reverse Transcription kit, 50 ng of random primers (both from Invitrogen, Life technologies, Carlsbad, California, USA) and 0.5 mM dNTP mixture (Bioline, London, UK). QPCR was done in a CFX96 real-time PCR detection system using 30 ng of cDNA, 1X iQ SYBR-green supermix (Biorad, Hercules, California, US) and primers at a final concentration of 2 mM. Cycling conditions included a pre-incubation step (95°C for 3 minutes), 40 cycles of amplification (95°C for 30 seconds, annealing temperature for 30 seconds and 72°C for 30 seconds) and a melting curve ( 95°C for 1 minute, 55°C for 1 minute, denaturation from 55°C to 95°C at 0.5°C/10 seconds increments). Each reaction was done in triplicate. All primers used are listed in Additional file 1: Table S8.

Expression values were normalised to the geometrical mean of housekeeping genes *RPL11*, *RPL30* and *RPS6* and fold-changes between treatments and controls were determined by the 2^-ΔΔCt^ method [57] as implemented in the SLqPCR Bioconductor package (http://www.bioconductor.org/packages/2.12/bioc/html/SLqPCR.html). The variance between sample groups was assessed through the Barlett test for variance. If variances could be assumed as equal between groups, significant differences were established using ANOVA followed by pairwise t-test. If variances could not be assumed as equal, significant differences were then assessed using a one way test for equal means followed by pairwise t-test. After multiple test correction by Benjamini and Hochberg method [54], results were considered significant at two levels: with adjusted p value lower or equal to 0.05 and with adjusted p value lower or equal to 0.08. All analysis was done using R v9 (http://www.r-project.org).

### Protein immunoblotting

Cells were lysed in urea/SDS buffer (6.7 M urea, 10 mM Tris-Cl pH 7.4, 10% glycerol, 1% SDS) supplemented with 1 mM dithiothreitol. Extracts were resolved by SDS-PAGE, electroblotted onto PVDF membranes (Millipore) and probed with primary antibodies followed by HRP-conjugated secondary antibodies. SuperSignal Chemiluminescent Substrates (Pierce) were used to visualize immunoreactive species. Antibodies used: anti-HIF1α 610959 from BD Biosciences, anti-HIF2α 190b from Peter Ratcliffe Lab, and anti-β-Actin/HPR ab49900 from Abcam.

### Short interfering RNA treatment of MCF-7 cells

RNA oligonucleotides (Ambion, Life technologies, Carlsbad, California, USA) were used for siRNA suppression of individual *HIF* family members (*HIF-1α*: sense, CUGAUGACCAGCAACUUGAtt; antisense, UCAAGUUGCUGGUCAUCAGtt. *HIF-2α*; sense, CAGCAUCUUUGAUAGCAGUtt; antisense, ACUGCUAUCAAAGAUGCUGtt). A set of oligonucleotides (Ambion, Life technologies, Carlsbad, California, USA) against a sequence from the *Drosophila melanogaster HIF* sequence, which lacks any substantial sequence similarity with human *HIF* or other genes, was used as a negative control (*D-HIF*: sense, CCUACAUCCCGAUCGAUGAtt; antisense, UCAUCGAUCGGGAUGUAGGtt) in one of the experiments. MCF-7 cells were seeded at 30% confluence and grown in normoxic conditions. Cells were transfected twice with 20 nmol/L of siRNA at 24 and 48 hours using OligofectAMINE (Invitrogen, Life technologies, Carlsbad, California, USA) according to the instructions of the manufacturer. At 55 hours, cells were exposed to 1% oxygen, and after an additional 16 hours, RNA was extracted. Each siRNA treatment was done in triplicate as well as the parallel control using OligofectAMINE reagent alone. The same protocol was used for the generation of protein extracts. We ensured that specific and substantial knockdown was occurring in MCF-7 cells under hypoxia by assaying HIF-α levels by immunoblotting (as previously described [26]).

### Statistical methods

A signature of 99 genes up-regulated under hypoxic conditions has been derived in head and neck tumors in vivo and it has been assessed for its prognostic value in a head and neck and breast cancer data set [31]. Here, this signature was used to calculate a hypoxic score for a breast cancer series using the gene expression profiles (GSE22220) published in [32]. The score is a measure of the level of expression of genes in the hypoxia signature: a high positive score reflects a higher than average expression of the genes in the hypoxia signature and indicates that the sample is hypoxic, a low negative score reflects a lower than average expression of the genes in the hypoxia signature and indicates that the sample is normoxic [31]. Correlation of microRNA expression from the same breast cancer series (GSE22220) and hypoxia score was assessed using Spearman’s rank tests. Statistical analyses were done using R.

## Abbreviations

AGO: Argonaute; ChIP: Chromatin immunoprecipitation; ChIP-seq: Chromatin immunoprecipitation sequencing; GSEA: Gene set enrichment analysis; HIF: Hypoxia inducible factor; HUVEC: Human umbilical vein endothelial cells; PASMC: Pulmonary artery smooth muscle cells; Pre-miRNA: Precursor microRNA; Pri-miRNA: Primary microRNA; qPCR: Real time PCR or quantitative PCR; RISC: RNA induced silencing complex; ROS: Reactive oxygen species; siRNA: Short interfering RNA; VHL: Von Hippel Lindau.

## Competing interests

The authors declare that they have no competing interests.

## Authors’ contributions

CC did the experimental work and generated the data (microRNA-seq, microRNA and gene expression microarray and qPCR data), participated in the analysis of the different datasets, integrated the results and drafted the manuscript. HKS, MR, JAGA, AGH and AJE participated in the analysis of the microRNA sequencing data. DRM provided the HIF ChIP-seq data and participated in the integration of this data to microRNA-seq data. HC provided directional ribo-depleted RNA-seq data and participated in the integration of this data to HIF ChIP data. YMT did the HIF protein immunoblots. FMB participated in the analysis of microRNA and gene expression microarray data and the correlations to hypoxia score. ALH participated in the design of the study and helped to draft the manuscript. JR conceived of the study, supervised the experiments and manuscript production. All authors read and approved the final manuscript.

## Supplementary Material

Additional file 1: Table S1 MicroRNA-seq analysis. Details are given on analysis output at different stages. **Table S2.** MicroRNAs detected in MCF-7 cells by microRNA-seq. **Table S3.** MicroRNAs commonly detected by microRNA-seq and Agilent microRNA arrays in MCF-7 cells. **Table S4.** MicroRNA-seq data for microRNAs within 50 kb distance of a HIF binding site. MicroRNA microarray data also included when available. **Table S5.** microRNAs encoded within a coding gene sequence. MicroRNA-seq and mRNA microarray expression of corresponding host gene are provided. **Table S6.** Pairs of microRNA and host genes with low coordination between their expression. Information included as in Table S5. Significant up- and down-regulations are highlighted in red and green, respectively. **Table S7.** Microarray data for genes involved in the microRNA processing. **Table S8.** Primers used in the qPCR analysis. **Table S9.** Comparison with previously published hypoxia studies. MicroRNAs reported to be hypoxia regulated in previous studies are included and the regulation is indicated as up (up-regulated) and down (down-regulated). Concerning our data, microRNAs found significantly regulated at each hypoxia time (16 h, 32 h and 48 h) as well as HIF binding sites identified are included. Hypoxic regulations determined by qPCR are indicated with*. **Table S10.** Differential expression of microRNA processing genes using data from published hypoxia studies. Significant changes are highlighted in red (for up-regulation) and green (for down-regulation). **Note for Table S2 to Table S7 and Table S10:** microRNAs or genes (Tables S7 and S10) are arranged in numerical order for ease of reading. Fold change (FC) in linear scale (hypoxia vs normoxia) and p-value before and after multiple test correction (adj.p-val) are shown for each microRNA and gene at each hypoxia time (only adj.p-val is shown in Table S10). Platform specified at the header when multiple data sets are displayed in the same table. Click here for file

Additional file 2: Figure S1 Validation of microRNA sequencing data by microRNA microarray. RNA samples were hybridised to Agilent microRNA arrays (3 biological replicates per condition). We found 228 microRNAs commonly detected between sequencing and microarray platforms. The fold-changes obtained for each microRNA in each platform have been compared for each hypoxia time and represented in scatter plots in log2 scale. MicroRNAs found significantly regulated by sequencing (adj.p-val < 0.05) are highlighted in colours: in blue if they are only significantly regulated in the given hypoxia time point or in red if they are as well significantly regulated in other time points. Solid colours represent microRNAs that were also found significant by microarray analysis (limma, adj.p-val < 0.05) in the particular hypoxia time point. The correlation (pearson) between fold-changes is 0.37 (p-val = 8.47e-09) in hypoxia 16 h, 0.27 (p-val = 2.85e-05) in hypoxia 32 h and 0.30 (p-val = 3.75e-06) in hypoxia 48 h. The correlation is better when only considering the microRNAs found signficantly regulated by sequencing: 0.43 (p-val = 0.0052) in hypoxia 32 h and 0.42 (p-val = 0.0019) in hypoxia 48 h. Click here for file

Additional file 3: Figure S2 Correlation between microRNA expression obtained from microarray data and corresponding host gene expression. Expression of genes hosting microRNAs was obtained from microarray data and expression of corresponding microRNAs was obtained from microRNA microarray data. The hypoxic regulation at 32 h and 48 h for both groups was then compared from two different perspectives. First, hosting genes were sorted in 3 groups at each time point: significantly down-regulated (down), not significantly regulated (unv) and significantly up-regulated (up). The fold-change distribution for each group of genes at 32 h and 48 h of hypoxia compared to normoxia is shown in boxplots (A). For each group of genes, the fold-change distribution of corresponding microRNAs at 32 h and 48 h compared to normoxia is also shown in boxplots for comparison (B). Second, microRNAs hosted within genes were sorted in 3 groups according to their hypoxic regulation at 32 h and 48 h: significantly down-regulated (down), not significantly regulated (unv) and significantly up-regulated (up). The fold-change distribution for each group of microRNAs at 32 h and 48 h of hypoxia compared to normoxia is shown in boxplots (C). For each group of microRNAs, the fold-change distribution of corresponding host genes at 32 h and 48 h compared to normoxia is also shown in boxplots for comparison (D). All fold-change distributions are shown in linear scale. Click here for file
