# Supplementary figures and images for: Integrated analysis of microRNA and mRNA expression and association with HIF binding reveals the complexity of microRNA expression regulation under hypoxia
Source: Mol Cancer. 2014 Feb 11;13:28. doi: 10.1186/1476-4598-13-28 (PMC3928101; doi:10.1186/1476-4598-13-28)

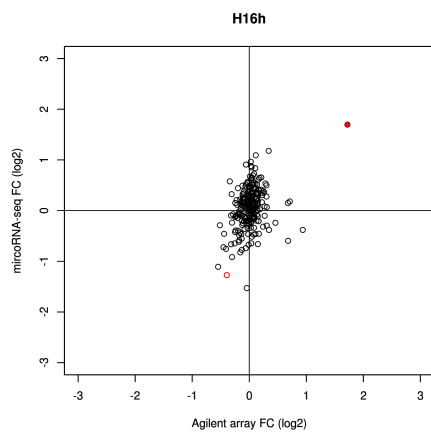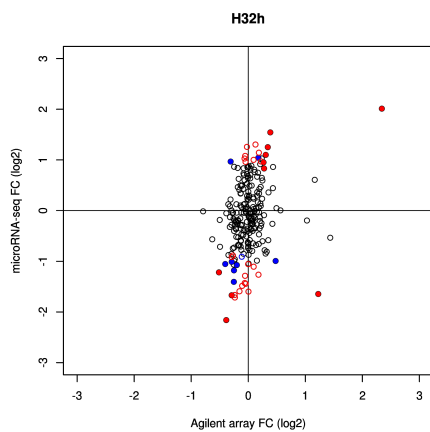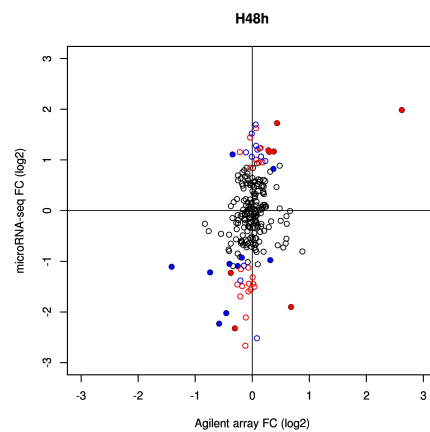

Supplement: Additional file 2: Figure S1 — Validation of microRNA sequencing data by microRNA microarray. RNA samples were hybridised to Agilent microRNA arrays (3 biological replicates per condition). We found 228 microRNAs commonly detected between sequencing and microarray platforms. The fold-changes obtained for each microRNA in each platform have been compared for each hypoxia time and represented in scatter plots in log2 scale. MicroRNAs found significantly regulated by sequencing (adj.p-val < 0.05) are highlighted in colours: in blue if they are only significantly regulated in the given hypoxia time point or in red if they are as well significantly regulated in other time points. Solid colours represent microRNAs that were also found significant by microarray analysis (limma, adj.p-val < 0.05) in the particular hypoxia time point. The correlation (pearson) between fold-changes is 0.37 (p-val = 8.47e-09) in hypoxia 16 h, 0.27 (p-val = 2.85e-05) in hypoxia 32 h and 0.30 (p-val = 3.75e-06) in hypoxia 48 h. The correlation is better when only considering the microRNAs found signficantly regulated by sequencing: 0.43 (p-val = 0.0052) in hypoxia 32 h and 0.42 (p-val = 0.0019) in hypoxia 48 h. [file 1476-4598-13-28-S2.pdf]

**A)**

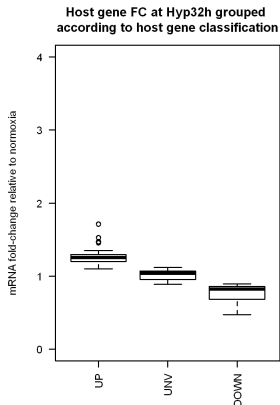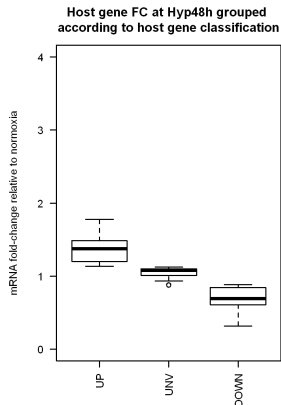

**B)**

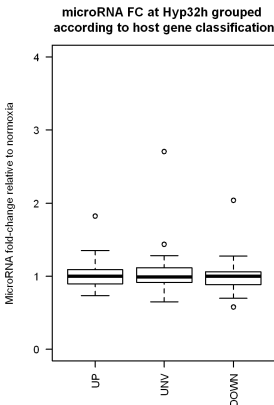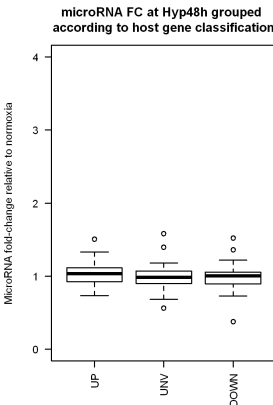

**C)**

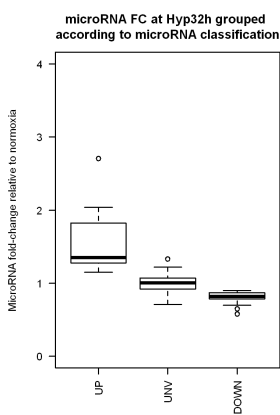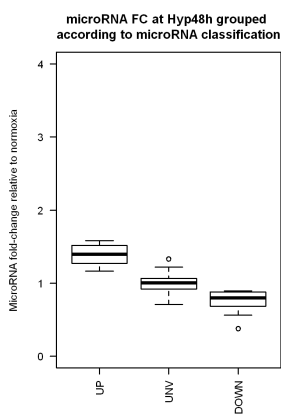

**D)**

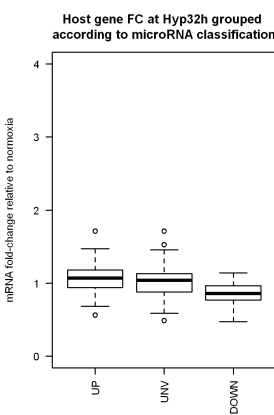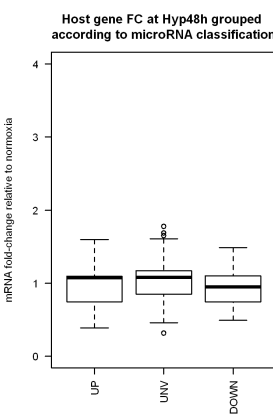

Supplement: Additional file 3: Figure S2 — Correlation between microRNA expression obtained from microarray data and corresponding host gene expression. Expression of genes hosting microRNAs was obtained from microarray data and expression of corresponding microRNAs was obtained from microRNA microarray data. The hypoxic regulation at 32 h and 48 h for both groups was then compared from two different perspectives. First, hosting genes were sorted in 3 groups at each time point: significantly down-regulated (down), not significantly regulated (unv) and significantly up-regulated (up). The fold-change distribution for each group of genes at 32 h and 48 h of hypoxia compared to normoxia is shown in boxplots (A). For each group of genes, the fold-change distribution of corresponding microRNAs at 32 h and 48 h compared to normoxia is also shown in boxplots for comparison (B). Second, microRNAs hosted within genes were sorted in 3 groups according to their hypoxic regulation at 32 h and 48 h: significantly down-regulated (down), not significantly regulated (unv) and significantly up-regulated (up). The fold-change distribution for each group of microRNAs at 32 h and 48 h of hypoxia compared to normoxia is shown in boxplots (C). For each group of microRNAs, the fold-change distribution of corresponding host genes at 32 h and 48 h compared to normoxia is also shown in boxplots for comparison (D). All fold-change distributions are shown in linear scale. [file 1476-4598-13-28-S3.pdf]
